# OICP: An Online Fast Registration Algorithm Based on Rigid Translation Applied to Wire Arc Additive Manufacturing of Mold Repair

**DOI:** 10.3390/ma14061563

**Published:** 2021-03-22

**Authors:** Ruibing Wu, Ziping Yu, Donghong Ding, Qinghua Lu, Zengxi Pan, Huijun Li

**Affiliations:** 1 School of Mechatronic Engineering and Automation, Foshan University, Foshan 528225, China; 2111851023@stu.fosu.edu.cn (R.W.); zy755@uowmail.edu.au (Z.Y.); qhlu@fosu.edu.cn (Q.L.); 2 School of Mechanical, Materials, and Mechatronics Engineering, Faculty of Engineering and Information Sciences, University of Wollongong, Northfield Ave., Wollongong, NSW 2500, Australia; zengxi@uow.edu.au (Z.P.); huijun@uow.edu.au (H.L.)

**Keywords:** mold repairing, wire arc additive manufacturing, 3D reconstruction, fast registration

## Abstract

As promising technology with low requirements and high depositing efficiency, Wire Arc Additive Manufacturing (WAAM) can significantly reduce the repair cost and improve the formation quality of molds. To further improve the accuracy of WAAM in repairing molds, the point cloud model that expresses the spatial distribution and surface characteristics of the mold is proposed. Since the mold has a large size, it is necessary to be scanned multiple times, resulting in multiple point cloud models. The point cloud registration, such as the Iterative Closest Point (ICP) algorithm, then plays the role of merging multiple point cloud models to reconstruct a complete data model. However, using the ICP algorithm to merge large point clouds with a low-overlap area is inefficient, time-consuming, and unsatisfactory. Therefore, this paper provides the improved Offset Iterative Closest Point (OICP) algorithm, which is an online fast registration algorithm suitable for intelligent WAAM mold repair technology. The practicality and reliability of the algorithm are illustrated by the comparison results with the standard ICP algorithm and the three-coordinate measuring instrument in the Experimental Setup Section. The results are that the OICP algorithm is feasible for registrations with low overlap rates. For an overlap rate lower than 60% in our experiments, the traditional ICP algorithm failed, while the Root Mean Square (RMS) error reached 0.1 mm, and the rotation error was within 0.5 degrees, indicating the improvement of the proposed OICP algorithm.

## 1. Introduction

In modern industry, molds can be used for the mass production of high-quality medium-to-large work pieces. However, under high strength and long-term working loads, the mold may become damaged, thereby reducing its reliability. The manufacturing process of the mold is very cumbersome and time-consuming, so once the mold has failed to continue the fabrication process, repairing it is the best solution. In this case, repairing faulty molds, prolonging their life, reducing production costs, and saving materials have become urgent problems for the entire mold industry [1].

The traditional mold repair method is through manual arc surfacing, which requires the welder to use carbon arc gouging to sink the mold surface of the faulty mold and then use manual arc welding to deposit the material into the cavity shape. Finally, the repaired mold undergoes corresponding heat treatment and mechanical processing to restore the size and performance of the standard mold [2,3]. This method has lower equipment and technology requirements, lower welding costs, and a flexible repair process. However, the shortcomings are also prominent. The accuracy of profiling welding for mold modeling can only depend on the experience of the welder, so if the quality of repair is not stable enough, it is far from meeting the needs of modern mold repair [4].

One possible solution to achieve a high-quality repair of the mold is to employ Additive Manufacturing (AM) [5] technologies that can directly build up near-net-shaped parts. Additive Manufacturing has excellent development potential in manufacturing complex structural parts, large size parts, or high cost parts. It is not constrained by complex conditions, such as processing tools, molds, or fixtures combined with the traditional subtractive processing. Regarding the AM of metal components, there are many techniques have been developed, such as selective laser sintering [6], direct metal deposition [7], electron beam freeform fabrication [8], and Wire Arc Additive Manufacturing (WAAM) [9].

WAAM is a technology that can offer a proper solution to these problems. It has been investigated and well developed over the last 30 years [10]. WAAM uses metal welding wire as the feedstock and electric arc as the heat source, making it a combination of welding and AM technology. It uses standard welding equipment (power source, welding torch, welding wire, and protective gas feeding systems). In recent years, WAAM has drawn increasing research interest due to its high deposition efficiency and low cost in producing large parts or complex structures. WAAM combines the additive manufacturing and surfacing repair process using machinery to complete labor-intensive and repetitive welding work, thereby ensuring the stability of welding quality that is not restricted by time, place, and environment. It is one of the most promising directions for automatic mold repair [3]. As one of the advanced welding processes, the use of Cold Metal Transfer technology (CMT) [11] exhibits the advantages of high quality, no splash, and low heat input, which can further improve the accuracy and practicability of repairing molds.

In the use of WAAM intelligent repair for molds, point cloud models can restore failed molds and provide visual repair solutions. The point cloud model contains a set of massive points that express the spatial distribution and surface characteristics of the target, which can accurately represent a 3D mesh model of a work piece [12]. Due to the limitations of 3D scanning technology and the high precision requirements of repaired models, it is usually necessary to capture multiple datasets from different angles, and each dataset is associated with the other coordinate system. Moreover, the mold used in the industry can be considerably large. Directly establishing one point cloud model of the entire work piece will result in extremely high data recording and processing difficulties. These data must be registered to reorganize multiple 3D datasets and align overlapping components. Therefore, surface registration is proposed to transform them into the same coordinate system in order to reconstruct the surface representing the original object or scene. It is also an integral part of the 3D acquisition pipeline and is the basis of computer vision, computer graphics, and reverse engineering [13].

With rapid advances in sensor technologies, it is now easy to capture depth data at high frame rates. However, the high acquisition rate comes at the cost of lower data quality [14]. The scanning results are often sparse and incomplete with image noise, so the further data consolidation stage is then processed. A standard solution is to accumulate multiple scans across time (e.g., using Kinect Fusion [15]). Such an approach performs local alignment using Iterative Closest Point (ICP) [16] technology and assumes the scene to be static and the subsequent scans to be closely aligned. Hence, for predictable accumulation results, the acquisition device must be moved slowly or rigged with reliable GPS tracking to assist in the initial positioning of the scans.

Unfortunately, the ICP requirement demands that the initial positions of the two -point clouds should not be too different and also need a high overlap area [12,17]. Using ICP to merge two large point clouds with a low overlap area (<60%) is inefficient, time -consuming, and unsatisfactory. 

In this paper, a WAAM of mold repair systems is proposed to realize online fast scanning and repairing. The fast registration method suitable for intelligent WAAM repair technology of molds is also employed, as detailed in Section 3. The proposed algorithm is named Offset ICP (OICP) since it is developed and optimized from the computational structure of a standard ICP. Its practicality for mold repair and the high computational accuracy for point cloud models with low overlapping areas are detailed in Section 4.

## 2. Registration Algorithm Development

### 2.1. The ICP Algorithm

ICP (Iterative Closest Point) [16] is a widely used algorithm for point cloud registration, but it has certain drawbacks, such as a small convergence basin and a high number of iteration steps needed until convergence is reached.

The basic principle of the ICP algorithm, according to certain constraints, is to find the closest Euclidean distance ($p_{i}$,$q_{i}$) as the corresponding point for each point *i* within the two sets of point clouds, “P’ and “Q”, to be matched. Furthermore, the least-squares method is used to iteratively calculate the optimal matching parameters in order to make the error function *E*(*R*,*t*) as small as possible, which is calculated by the following:

(1) $$E(R,t)=\frac{1}{n}{\sum_{i=1}^{n}\left\|q_{i}-\left(Rp_{i}+t\right)\right\|}^{2}$$

where *R* is the rotation matrix, and *t* is the translation matrix.

Many reasons affect the matching of the ICP algorithm. The ICP algorithm relies heavily on the initial registration position. It requires the initial positions (overlap area) of point clouds to be sufficiently close enough. As the overlap area decreases, the reliability of the final model gradually decreases. If the overlap area of the two-point clouds, “P” and “Q”, is too low, the ICP algorithm will fail and cannot obtain a robust registration point cloud [17].

Another drawback of the ICP algorithm is that it is not suitable for registering enormous point cloud data. The scanner used in the paper has high accuracy, and the number of point cloud samples obtained per scan is about 600,000 [18] to solve the above-mentioned problems. Therefore, a robust variant algorithm is urgently needed to register multiple point clouds based on the ICP algorithm.

### 2.2. The OICP Algorithm

In order to reduce the impact of the low overlap area on the point cloud registration, improve calculation speed and accuracy of the reconstruction and ultimately realize fast online scanning of molds, an alternative method can be adopted once the point-to-point correspondences of the point cloud are entirely known in both datasets [13].

In WAAM, the work piece is fixed on the worktable during the whole manufacturing process, so the initial position of each point cloud can be calibrated. Therefore, the transformation between every two adjacent point clouds can be defined as the offset transformation on the *x*-axis, and the moving machine obtains the offset (*t_x_*). The ultimate goal of point cloud registration is to unify two, or more, point cloud datasets in different coordinate systems into the same reference coordinate system by transforming rigid rotation and linear translation. The traditional ICP algorithm objective is to obtain the final rigid transformation matrix through continuous iteration. The form of describing the rigid transformation matrix is 4 × 4, defined as

(2) $$H=\left[\begin{array}{cccc} a_{11} & a_{12} & a_{13} & v_{x}\\ a_{21} & a_{22} & a_{23} & v_{y}\\ a_{31} & a_{32} & a_{33} & v_{z}\\ t_{x} & t_{y} & t_{z} & s \end{array}\right]$$

(3) $$H=\left[\begin{array}{cc} R_{3\times 3} & V\\ T_{1\times 3} & S \end{array}\right]$$

where *R* represents the rotation matrix, *T* represents the translation vector, *V* represents the perspective transformation vector, and *S* represents the overall scale factor. As the point cloud data obtained from a series of pictures only have rotation and translation transformations and there is no deformation, *V* is set as a zero vector and the scale factor *S* = 1.

(4) $$R_{3\times 3}=\left[\begin{array}{ccc} 1 & 0 & 0\\ 0 & \cos\alpha & \sin\alpha \\ 0 & -\sin\alpha & \cos\alpha \end{array}\right]\left[\begin{array}{ccc} \cos\beta & 0 & -\sin\beta \\ 0 & 1 & 0\\ \sin\beta & 0 & \cos\beta \end{array}\right]\left[\begin{array}{ccc} \cos\gamma & \sin\gamma & 0\\ -\sin\gamma & \cos\gamma & 0\\ 0 & 0 & 1 \end{array}\right]$$

(5) $$T_{1\times 3}=\left[\begin{array}{ccc} t_{x} & t_{y} & t_{z} \end{array}\right]$$

where *α*, *β*, and *γ* represent the rotation angle of the point along the *x*, *y*, *z* axes, respectively, and t*_x_*, t*_y_*, and t*_z_* represent the translation of the point along the *x*, *y*, and *z* axes, respectively.

In this experiment, since all point clouds are almost aligned, there is only an *x*-axis translation between two adjacent point clouds, defined as

(6) $$H=\left[\begin{array}{cccc} 1 & 0 & 0 & 0\\ 0 & 1 & 0 & 0\\ 0 & 0 & 1 & 0\\ t_{x} & 0 & 0 & 1 \end{array}\right]$$

When performing coordinate transformation of points *X* and *X’* in two different coordinate systems, the transformation can be achieved through the following formula:

(7) $$X^{\prime }=HX$$

**Figure 1:**
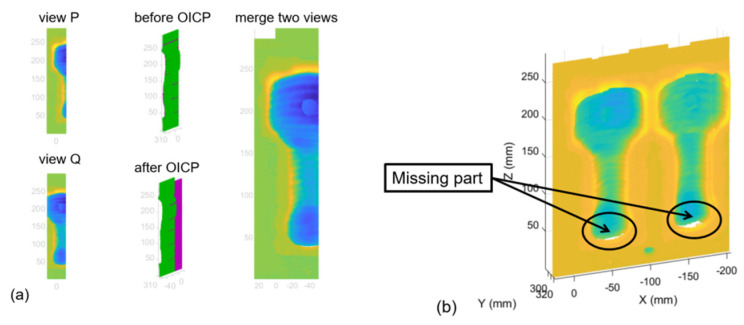
Merging process of the Offset Iterative Closest Point (OICP) algorithm. (**a**) Rigid transformation and merging of two point clouds; (**b**) rigid transformation and merging of multiple point clouds and missing part.

Thus, the first step of the registration algorithm is to set a proper scan path with the *x*-axis to offset and return an *H* rotation matrix that can be used to directly transform the source point cloud into the overlap area of the target. Figure 1a shows the two adjacent point clouds merged through a translation matrix. Figure 1b shows that the point cloud model loses detail in the same coordinate system as the point acquisition process. Therefore, in that case, it is necessary to rotate the scanning coordinate system to obtain the missing part of the point cloud data.

**Figure 2:**
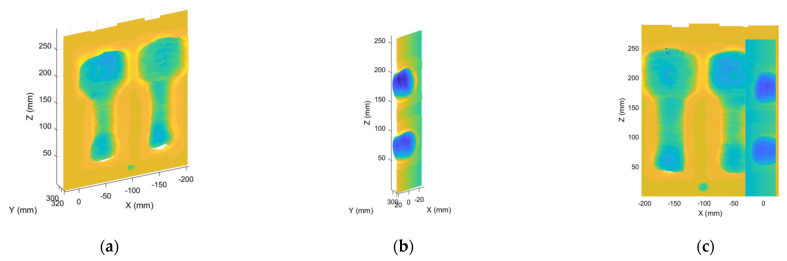
The relative position of the missing point cloud part. (**a**) Initial registration image; (**b**) the missing part; (**c**) initial relative position.

**Figure 3:**
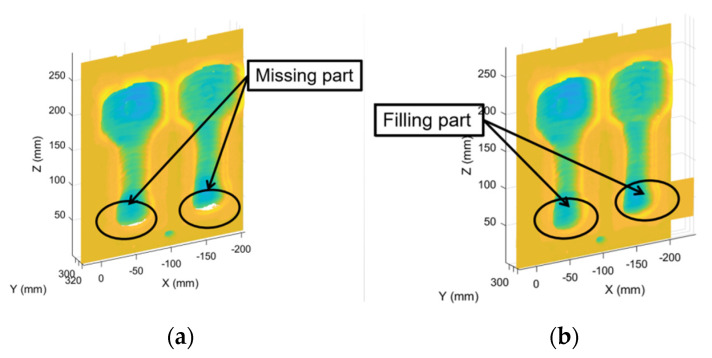
OICP algorithm to fill in the missing part. (**a**) Initial registration image; (**b**) final registration image.

The next step of registration is to fill in the missing part of the point cloud. During the calculation, the rotation in the *x*-axis (*α*) and *y*-axis (*β*) is known, so the initial rotation matrix is obtained. After rotating the missing point cloud and the initial registration point cloud in the same direction, since the overlap rate is already high at this time, the ICP algorithm is then used to obtain the final rigid transformation matrix and to register the final point cloud, which is calculated by

(8) $$R_{2}=\left[\begin{array}{ccc} 1 & 0 & 0\\ 0 & \cos\alpha & \sin\alpha \\ 0 & -\sin\alpha & \cos\alpha \end{array}\right]\left[\begin{array}{ccc} \cos\beta & 0 & -\sin\beta \\ 0 & 1 & 0\\ \sin\beta & 0 & \cos\beta \end{array}\right]=\left[\begin{array}{ccc} \cos\beta & 0 & -\sin\beta \\ \sin\alpha \sin\beta & \cos\alpha & \sin\alpha \sin\beta \\ \cos\alpha \sin\beta & -\sin\alpha & \cos\alpha \cos\beta \end{array}\right]$$

(9) $$H_{2}=\left[\begin{array}{cccc} \cos\beta & 0 & -\sin\beta & 0\\ \sin\alpha \sin\beta & \cos\alpha & \sin\alpha \cos\beta & 0\\ \cos\alpha \sin\beta & -\sin\alpha & \cos\alpha \cos\beta & 0\\ t_{x} & t_{y} & t_{z} & 1 \end{array}\right]$$

(10) $$Y^{\prime }=H_{2}Y$$

where $H_{2}$ is the initial rotation and translation matrix moving the missing point cloud M to the same position as the current registration point cloud N. After the coarse registration, the ICP algorithm is employed to obtain the optimal rotation and translation matrix iteratively and realize the missing point cloud registration, as shown in Figure 2 and Figure 3.

## 3. Experimental Setup

### 3.1. Point Cloud Data Acquisition

**Figure 4:**
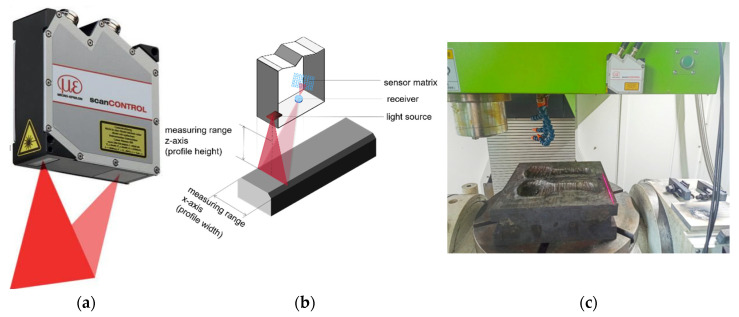
The device used for the point cloud acquisition. (**a**) The scanCONTROL 2900 laser scanner and (**b**) its measuring principle; (**c**) the position of the scanner used in the experiment.

The scanCONTROL 2900 laser scanner, as shown in Figure 4a, was used in this paper to adopt the laser triangle reflection principle to collect the two-dimensional contour information of different surface materials. Through a select lens group, the laser beam was magnified to form a static laser line and projected onto the surface of the measured object. The laser line formed a diffuse reflection on the surface of the measured object, and the reflected light passed through a high-quality optical system and was then projected onto the sensitive photosensitive matrix, as shown in Figure 4b.

In addition to the distance information (*z*-axis) from the sensor to the measured surface, the controller can also calculate the position information along the laser line (*x*-axis) from the image information. In a two-dimensional coordinate system centered on the sensor, the scanner measures and outputs a set of two-dimensional coordinate values. Placing the scanner and the measured object vertically and moving the measured object or scanner probe provide a number of three-dimensional measurement values.

The mold was processed on the Z-YANG V15L (Siemens 840D) three-axis Computer Numerical Control (CNC) machine, and the scanning direction was set along the *y*-axis. The scanner was used to measure and fill the mold in blocks and then to collect point cloud data and store them in an ordered list.

Since the scanner sensor can only obtain *x*-axis coordinates and *z*-axis coordinates as the scanning direction is determined, the *y*-axis coordinate needs to be added by:

(11) y=y+a×i (i=1,2,3,…,n)

(12) $$a=\frac{v}{60f}$$

where *i* represents the *i*-th point in the list, and *a* represents the step of obtaining the coordinate point, which is determined by the CNC machine bed motion speed *v* and the data scanning frequency *f*.

### 3.2. WAAM Mold Repair System

**Figure 5:**
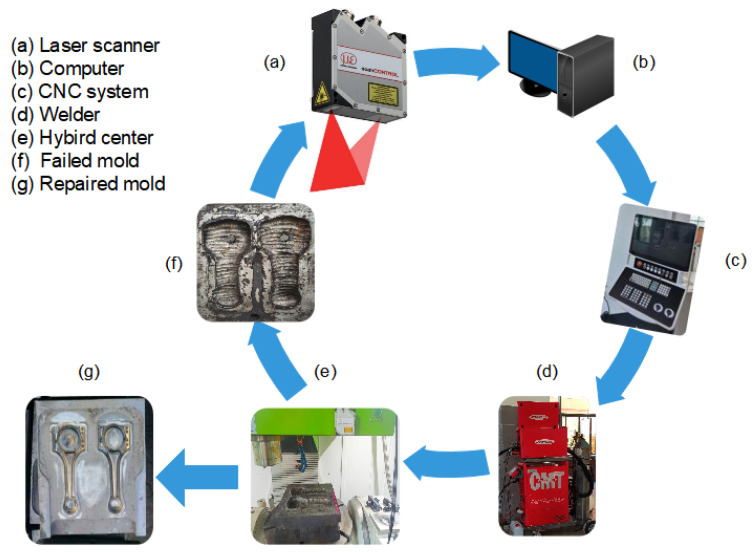
The Wire Arc Additive Manufacturing (WAAM) of the mold repair system based on a 3D profile laser scanner.

Figure 5 shows the WAAM of the mold repair system established based on the 3D profile laser scanner. The scanner (a), fixed on the CNC machine tool, was used to scan a part of the mold surface and input the generated point cloud data to the computer for data processing (b). After multiple scans to obtain point cloud models of the mold at different positions, the OICP algorithm was then used to merge these data points into a complete and reliable mold visualization 3D model. The CNC machine-bed provides linear motion in the *x*, *y*, and *z* directions to move the scanner and perform manufacturing tasks of the mold repair. To obtain complete point cloud datasets, the mold was loaded on the rotating table for the *x*-axis and *z*-axis rotation (c). The welding equipment employed was the TransPuls Synergic 5000 CMT welder (d). To one side of the spindle, the welding torch was mounted orthogonally so that it could perform with the CNC machine. The use of CNC machines can simultaneously perform additive and subtractive manufacturing processes (e). This hybrid processing method directly enables the WAAM process of repairing the mold and performs post-processing on its surface inside the machine, thus significantly improving the processing efficiency. The proposed mold repair system achieved highly repeatable results. After repairing one mold (g), the next failed mold (f) can be immediately inserted into the machine to start the new repair process.

**Figure 6:**
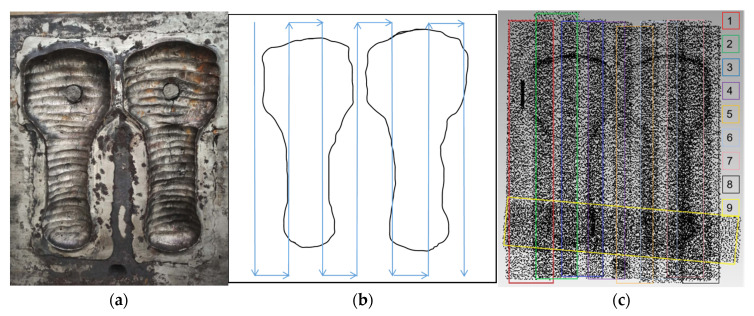
The point cloud model acquisition process. (**a**) Import failed mold; (**b**) path planning diagram; (**c**) point cloud data diagram after the OICP registration.

To establish point cloud models, a reasonable scan path needs to be determined first so as to ensure that each time movement path has the same scan range. The displacement of the CNC machine tool was increased step-by-step in the *x*-axis direction at a low speed to obtain multiple sets of scan results, as shown in Figure 6b. The point cloud registration was realized after the proposed OICP registration, as shown in Figure 6c. The experimental results in Section 4 prove that the OICP can also be completed quickly for point cloud registration with a low overlap (<60%) and high accuracy, which is suitable for the reverse work of WAAM mold repair [9].

## 4. Experimental Result and Discussion

During the experiment, after scanning the mold to generate multiple sets of point cloud models, the method of Pomerleau [19] was then employed to reduce the sampling rate of the enormous point cloud data, thereby improving computational efficiency. Moreover, the method of Rusu [20] was used for filtering in the nearest neighbor and distance.

The proposed OICP approach was first evaluated in terms of computation time improvement compared to the ICP algorithm at different overlap rates, and we finally proved its accuracy by comparing the results with the three-coordinate measuring instrument. The accuracy of other devices and the analysis of the advantages and disadvantages of the different instruments were also tested.

**Figure 7:**
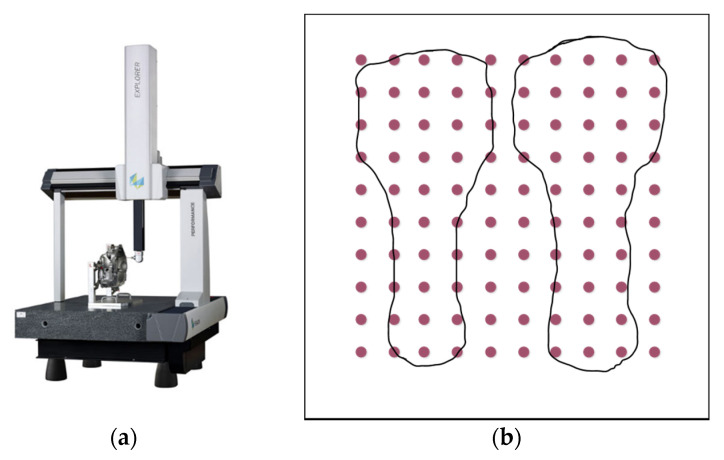
(**a**) Three-coordinate measuring instrument (Hexagon); (**b**) schematic diagram of 10 × 10 point cloud matrix.

It is worth noting that the three-coordinate measuring instrument has the advantages of extensive measuring range, high precision, and high efficiency. Therefore, Hexagon’s three-coordinate measuring machine is reliable for verifying the accuracy of the registered point cloud and the rationality and usability of the algorithm. Figure 7 is a schematic diagram of the position of the measuring point on the mold using a three-coordinate measuring machine, and the stylus is a 2 mm ruby probe. The aim of this experiment was to verify the global optimality of the OICP algorithm and to compare that with the standard ICP. One hundred tests were performed on random points. In each test, 100 model points were randomly drawn from the uniform distribution in [−1, 1, 3]; rotation and translation were randomly drawn within ±100 degrees and ±0.5, respectively, and then applied to the model points to generate data points; zero-mean Gaussian noise was added to the points; ICP was initialized with identity rotation and zero translation.

**Figure 8:**
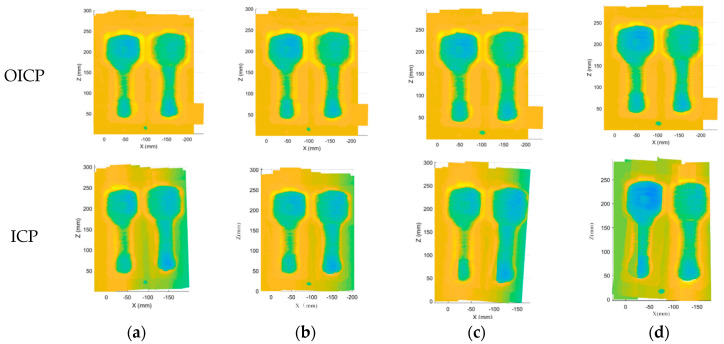
The effect of point cloud registration of ICP and OICP at a high overlap rate (≥60%): overlap = (**a**) 90%; (**b**) 80%; (**c**) 70%; (**d**) 60%.

**Figure 9:**
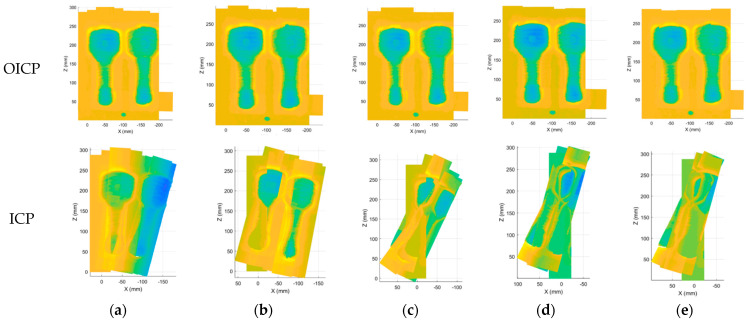
The effect of point cloud registration of ICP and OICP at a low overlap rate (<60%): overlap = (**a**) 50%; (**b**) 40%; (**c**) 30%; (**d**) 20%; (**e**) 10%.

**Figure 10:**
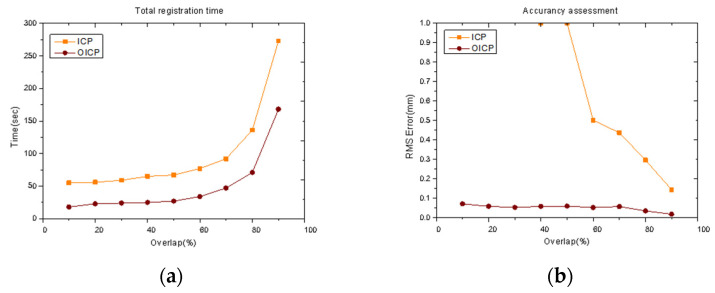
(**a**) Total time (s) needed for matching at different overlap rates; (**b**) the registration error based on the comparison with the three-coordinate measuring instrument.

In Figure 8 and Figure 9, practical examples show the effect of point cloud registration of ICP and OICP at various overlap rates. The OICP remained much more robust than the classic ICP in such low-overlap cases. When the overlap rate was less than 60%, the ICP was unable to register two adjacent point clouds, and when multiple point clouds were registered, the errors continued to add up, and only the wrong point cloud was obtained. Figure 10 shows the impact of different overlap rates and reports the total time to extract the pairs and build the congruent set and analyze it, both with ICP and OICP. The curve shows that the OICP is almost twice as fast as the ICP, with better robustness and more accurate point cloud data.

**Figure 11:**
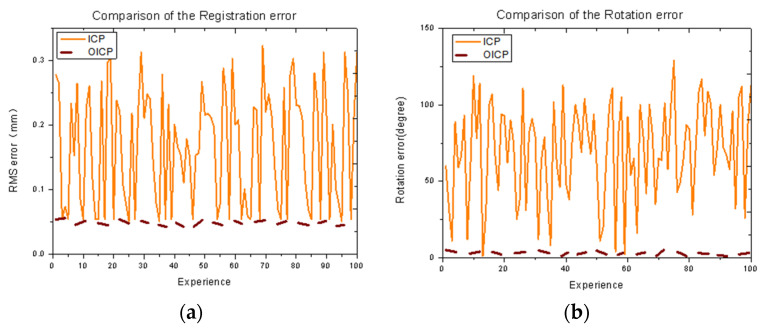
Comparison of the (**a**) registration error and (**b**) rotation error on random points by OICP method versus ICP initialized with identity rotation and zero translation. Ground truth rotation and translation lie randomly within ±100 degrees and ±0.5, respectively (point cloud overlap = 70%).

Moreover, the accuracy of the OICP was not affected by the overlap, which means that it is unnecessary to scan the failed mold multiple times in the future practice process. However, it is necessary to ensure that the mold is entirely scanned under the same overlap rate. Figure 11 shows the final reported registration error and rotation errors for the 100 runs [21,22]. The OICP always outperformed the classic ICP in terms of having a consistently lower residual error. Hence, the optimality is confirmed.

**Figure 12:**
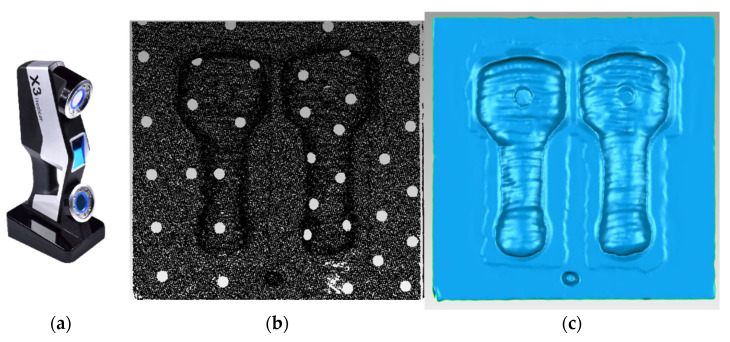
(**a**) Hand-held scanner (Freescan X3); (**b**) point cloud; (**c**) outliers removed and hole filled using Geomagic studio.

Simultaneously, a hand-held 3D scanner (Freescan X3 from Ten Youn) was used to measure the mold, as shown in Figure 12. The hand-held scanner has a fast measurement speed and high accuracy but a high price [23]. It has supporting software that can directly generate 3D point clouds, but it needs to paste the marker points on the mold, which is not suitable for real-time monitoring of mold WAAM repair.

**Figure 13:**
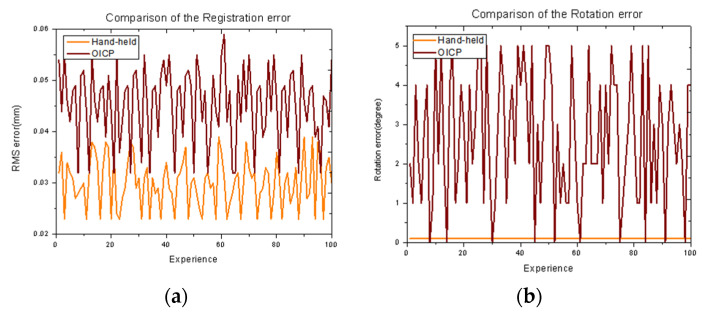
Comparison of the (**a**) registration error and (**b**) rotation error on random points by our OICP method and hand-held 3D scanner point cloud data. Ground truth rotation and translation lie randomly within ±5 degrees and ±0.5, respectively (point cloud overlap = 70%).

Figure 13 shows that the point cloud data obtained by the hand-held 3D scanner are better than that obtained by registering multiple point clouds using the OICP algorithm. One of the reasons is that the hand-held 3D scanner performs a one-time scan, and there are denoise algorithms [24] in the supporting software, while the error of point cloud registration continues to be superimposed. When using the OICP multiple times for registration in order to obtain a large object, registration error and rotation error will continue to add up.

When the point clouds obtained by three different measuring instruments are placed together and analyzed, although the accuracy of the hand-held 3D measuring instrument and the 3D profile laser scanner is already high, there is still a small gap with the point cloud data obtained by the 3D measuring instrument [25]. Nonetheless, the hand-held scanner with one scan can provide the best solution.

## 5. Conclusions

The OICP presented in this paper is a real-time fast registration algorithm based on rigid translation applied to WAAM mold repair, i.e., aligning scan pairs in specific poses for the surface. The algorithm is particularly useful in cases of low overlap across scans and is also time-saving. It is worth noting that even if the initial scan position is closely aligned, prior methods (like ICP) still fail in cases of low overlap, but OICP continues to work. The algorithm is tested in various scenarios and compared with competing alternatives, both in terms of speed and accuracy.

The proposed OICP algorithm is very suitable for the preliminary reverse work of mold repair based on WAAM. In the future, a complete repair system can be continually built with the algorithm applied to real-time monitoring of the additive manufacturing process. The algorithm will help the mold repair process, shorten repair time, and improve repair accuracy and quality.

## Data Availability

The data presented in this study are available on request from the corresponding author.
